# Case report: a synonymous *VHL* mutation (c.414A > G, p.Pro138Pro) causes pathogenic familial hemangioblastoma through dysregulated splicing

**DOI:** 10.1186/s12881-020-0976-7

**Published:** 2020-02-27

**Authors:** Fang Liu, Barbara Calhoun, Md. Suhail Alam, Miaomiao Sun, Xuechun Wang, Chao Zhang, Kasturi Haldar, Xin Lu

**Affiliations:** 1 Boler-Parseghian Center for Rare and Neglected Diseases, University of Notre Dame, Notre Dame, IN 46556 USA; 2 Department of Biological Sciences, University of Notre Dame, Notre Dame, IN 46556 USA; 3 Translational Medical Center for Stem Cell Therapy and Institute for Regenerative Medicine, Shanghai East Hospital, Shanghai Key Laboratory of Signaling and Disease Research, School of Life Sciences and Technology, Tongji University, Shanghai, 200092 China; 4 Harper Cancer Research Institute, University of Notre Dame, Notre Dame, IN 46556 USA; 5 Tumor Microenvironment and Metastasis Program, Indiana University Melvin and Bren Simon Cancer Center, Indianapolis, IN 46202 USA

**Keywords:** von Hippel-Lindau disease, Hemangioblastoma, Pheochromocytoma, Synonymous mutation, Silent mutation, Alternative splicing, Skin fibroblast, *VHL*, pVHL, Pro138Pro

## Abstract

**Background:**

von Hippel-Lindau (VHL) disease is a familial neoplasia syndrome that results from the germline mutation of *VHL*. Pathogenic *VHL* mutations include deletion, frameshift, nonsense and missense mutations. Synonymous mutations are expected to be phenotypically silent and their role in VHL disease remains poorly understood.

**Case presentation:**

We report a Caucasian male with a family history of pheochromocytoma and the synonymous *VHL* mutation c.414A > G (p.Pro138Pro). At 47-years, MRI revealed pheochromocytoma in the left adrenal gland and hemangioblastomas in the spine and brain. Pheochromocytoma was treated by adrenalectomy. Radiotherapy, followed by craniotomy and resection were needed to reduce hemangioblastomas to residual lesions. Two of three of the proband’s children inherited the mutation and both presented with retinal hemangioblastomas without pheochromocytoma at age 7: one twin needed four laser treatments. Primary skin fibroblasts carrying the heterozygous mutation or wild type *VHL* were established from the family. Mutant fibroblasts downregulated full-length *VHL* mRNA and protein, and upregulated the short VHL mRNA isoform (a result of exon 2 skipping in splicing) at the mRNA level but not at the protein level.

**Conclusions:**

Our study shows that the synonymous *VHL* mutation c.414A > G can within 7 years induce pediatric retinal hemangioblastoma in absence of pheochromocytoma. This highlights the need to include splicing-altering synonymous mutations into the screening for VHL disease. This is also the first report on detecting and validating a synonymous *VHL* mutation using patient-derived fibroblasts. The mutation c.414A > G translates to p.Pro138Pro, yet it is not functionally silent, because it causes aberrant splicing by skipping exon 2. The reduced but not completely abolished pVHL protein in a loss-of-heterozygosity genetic backdrop may underlie the etiology of VHL disease.

## Background

von Hippel-Lindau (VHL) disease is a rare autosomal dominant neoplasia syndrome affecting 1 in 36,000 births. Germline mutations in the *VHL* gene lead to the development of benign or malignant tumors in many organ systems [1–3]. Affected individuals have significantly heightened chance of developing lesions in the central nervous system (CNS) including hemangioblastoma (HGB) of cerebellum, spinal cord, brainstem and retina, as well as visceral tumors such as pheochromocytoma (PHEO), renal cell carcinoma (RCC), and pancreatic neuroendocrine tumors [1, 4, 5]. VHL disease has over 90% penetrance by 65 years of age [6]. The main causes of death are complications linked to RCC and CNS-HGB [6, 7]. VHL disease has a characteristic genotype-phenotype correlation: Type 1 has a very low risk of PHEO and is most commonly caused by *VHL* exon deletion, truncation, frameshift and nonsense mutations; Type 2 has a higher risk of PHEO and is characterized by *VHL* missense mutations [1, 2, 5]. Type 2 is further categorized into 2A (low risk of RCC), 2B (high risk of RCC), and 2C (only PHEO) [1, 2, 5]. VHL-associated tumors frequently lose the function of the remaining wild-type *VHL* allele in the process called loss of heterozygosity (LOH) [1, 2].

The protein pVHL is the substrate recognition unit of the E3 ubiquitin ligase complex composed of Elongin C, Elongin B, Cul2, and Rbx1. The complex targets hypoxia-inducible factor α (HIF1α and HIF2α) for degradation in normoxic conditions. In the absence of pVHL, HIFα is stabilized and translocated to the nucleus to activate transcription of target genes, many of which regulate tumor-promoting processes [1, 2]. pVHL also has HIF-independent functions [8–10].

Synonymous mutations are commonly referred to as silent mutations, because they are not expected to alter the function of encoded proteins. However, increasing evidence indicates that synonymous mutations may not be merely passenger events; instead, they can actively contribute to human cancers, often through alternation of pre-mRNA splicing [11–14]. For example, recurrent synonymous mutations in the tumor suppressor gene *TP53* were found to impair the wild type splice sites and activate cryptic splice sites [11]. *VHL* gene produces two protein-coding transcripts, the longer isoform encompasses exons 1, 2 and 3 (E1E2E3) whereas the shorter one lacks exon 2 (E1E3). E1E2E3 encodes a longer protein of 213 amino acids (pVHL_213_) and a shorter protein of 160 amino acids (pVHL_160_) due to translation initiation from an internal start site [15]. Both pVHL_213_ and pVHL_160_ are functional tumor suppressors [15]. E1E3 encodes a protein of 172 amino acids (pVHL_172_) with generally low expression abundance and a possible lack of the tumor suppressor function due to the disruption of the HIF-binding domain [16]. Recent studies suggest that synonymous mutations of *VHL* can also lead to dysregulated splicing [17, 18]. However, the clinical and molecular evidence to support the role of synonymous mutations in VHL disease is still very limited. Here, we provide an independent line of evidence to demonstrate that a synonymous mutation in exon 2 of *VHL* that shifts the pattern of splicing and expression of *VHL* at the cellular level is pathogenic to cause HGB both with and without PHEO.

## Case presentation

**Fig. 1:**
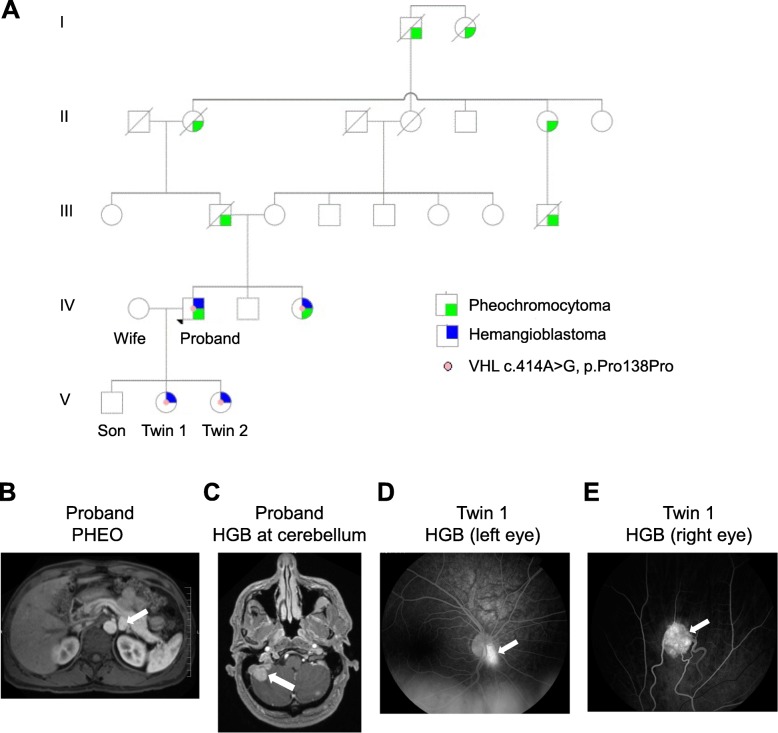
Clinical findings of the VHL disease family. (**a**) Five-generation pedigree of the patient family shows that the synonymous *VHL* variant c.414A > G segregates with affected family members. (**b**) T2-weighted MRI shows PHEO in the left adrenal gland of the proband. (**c**) Axial T1-weighted post contrast MRI shows the HGB in the right cerebellum of the proband. (D-E) Fluorescein angiography shows the retinal HGB in the left and right eye of Twin 1. In all images, white arrows point to the tumors

The proband presented here was a 41-year-old asymptomatic Caucasian male visiting his family physician for genetic testing of VHL disease. The proband’s sister was diagnosed with VHL disease after experiencing multiple tumors including paraganglioma of left carotid, spinal neuroma, bilateral adrenal PHEO and a skull based tumor with intracranial extension. Review of the family history identified that the proband’s father, paternal grandmother and paternal great grandfather all had clinical history of PHEO (Fig. 1a).

The proband’s test results revealed a heterozygous mutation from A to G at the nucleotide position 414 in the coding sequence of *VHL* transcript NM_000551, which surprisingly is a synonymous mutation that does not change the coded amino acid (p.Pro138Pro) of the protein pVHL. His clinical record is summarized in **Supplementary Table** **S1**. Six years after diagnosis, proband remained asymptomatic and at age of 47 he decided to take magnetic resonance imaging (MRI) of the brain, abdomen, pelvis and cervical, thoracic and lumbar spine. MRI revealed a PHEO in the left adrenal gland (Fig. 1b), five small HGB along the spine at C2, T12, L1 and L2 and multiple lesions within the posterior fossa of the brain. The patient underwent laproscopic adrenalectomy 2 months later to remove the PHEO without complications. Further evaluation of the brain tumors identified two large tumors in the right cerebellum (9 mm and 17 mm) (Fig. 1C) and a 4 mm ill-defined left posterior cerebellar enhancement, along with mild edema and displacement of the right cerebellar tonsil. The patient underwent gamma knife radiotherapy to the brain tumors and was re-evaluated. Repeat MRI of the brain showed that the prior right cerebellar tumors had evolved into one large conglomerate tumor (22x25x28mm), edema had increased and spread to majority of right cerebellar hemisphere. The right cerebellar horn was further displaced and the patient developed hydrocephalus with trans ependymal flow of cerebrospinal fluid and mass-effect on the inferior 4th ventricle. Subsequently, the patient underwent a posterior fossa craniotomy with resection of the large HGB. Postoperative MRI of the brain revealed resolution of the hydrocephalus. Repeat MRI in 3-month intervals (in 10/2008 and 01/2009) revealed normal brain without recurrence of tumors. From 2013 to 2019 small tumors of spine and cerebellar hemisphere persisted but remained unchanged. The patient is now 57 years old**.**

The proband’s wife has wild type *VHL*, and together they have three children: a son and younger fraternal twin daughters. Children underwent genetic testing and confirmed that the son had wild type *VHL* and the twins both inherited the c.414A > G mutation. The twins were diagnosed in 2012 at age 4. Subsequently, both began annual ophthalmology, endocrinology and otology screening along with routine physical exams. During an ophthalmology consult in 2015 (age 7), Twin 1 was noted to have a retinal HGB in both eyes: the left eye HGB was surrounded by sub-retinal fluid (Fig. 1d), and a small intra-retinal HGB of the right eye was located in close proximity to the optic nerve and therefore not operated on (Fig. 1e). Her left eye was treated with laser photocoagulation to dry the fluid and stabilize the fundus. Two months later, evaluation of the left eye revealed a residual retinal HGB (2.5 mm), which was treated with laser again. The blood flow to the tumor persisted and was treated two more times with laser on an outpatient basis to completely resolve the left eye HGB. Right eye HGB has remained unchanged. Twin 1 receives ophthalmologic exams three times a year to monitor the eye tumors along with brain, spine and abdominal MRI screenings. No other tumors have been found.

Twin 2 also underwent ophthalmology, endocrinology and hearing screening to identify any tumors and symptoms. In 2015, at age 7, a very small retinal HGB was identified in the central vision of the left eye. Due to its size and delicate surgical location, physicians decided to continue to monitor twice yearly. There is currently no fluid accumulation within the eye and the patient does not complain of any visual disturbances. The right eye is clear. Yearly MRI’s of the brain, spine and abdomen have all been negative for tumors. The twins were 11 years old in June 2019.

**Fig. 2:**
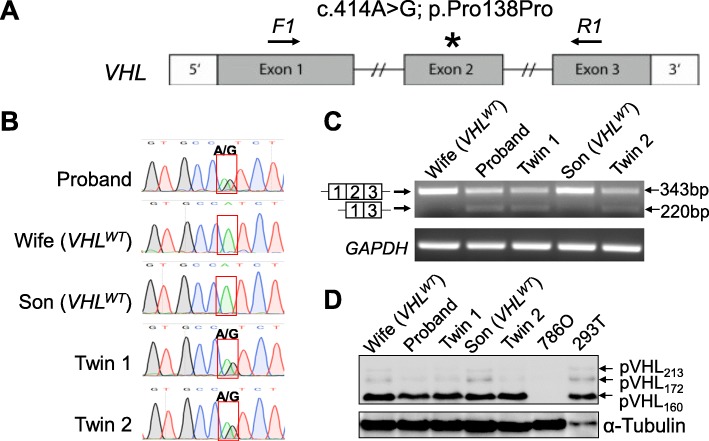
Genetic and expression analysis of synonymous mutations c.414A > G using primary fibroblasts. (**a)** Schematic of the exon structures of human *VHL* gene with the positions of the mutation and two RT-PCR primers indicated. (**b**) Chromatogram tracks showing the DNA sequence near the mutation site for each of the five fibroblast cell lines established from the patient family. (**c**) RT-PCR bands amplified from the cDNA of E1E2E3 and E1E3 transcripts using mRNA extracted from fibroblast cells. GAPDH was used as an internal control. (**d**) Western blot showing the three pVHL isoforms in the five fibroblast cell lines. RCC cell line 786O (VHL-null) and HEK293T (VHL-intact) were used as control for pVHL detection. α-Tubulin was used as loading control. VHL antibody is from Cell Signaling (cat#68547)

To confirm the synonymous mutation c.414A > G (Fig. 2a) and examine the mechanism how it causes VHL disease, we established primary skin fibroblast cell lines from the skin biopsies of the 5 individuals of the proband’s family: Proband, his wife, the son and the twin daughters. The fibroblasts were established as described [19] and cultured in DMEM, 10% fetal bovine serum and 1X Penicillin-Streptomycin, and all experiments using them were performed with early passages (< 8 passages). Genomic DNA was extracted from the fibroblast cells. The *VHL* exons were amplified using intronic primer pairs flanking each exon. The three exons of the *VHL* gene were determined through Sanger sequencing. The c.414A > G mutation was confirmed for the carriers (Fig. 2b). To examine whether this mutation affects splicing and gene expression, we first performed Reverse Transcriptase PCR (RT-PCR) on the 5 fibroblast cell lines using primers that locate in Exon 1 (F1: 5′-GCGTCGTGCTGCCCGTATG-3′) and Exon 3 (R1: 5′-TTCTGCACATTTGGGTGGTCTT-3′) of *VHL* transcript (shown schematically in Fig. 2a). We saw a significant change in the pattern of expressed *VHL* transcripts, with a higher expression level of the E1E3 mRNA at the expense of lower level of E1E2E3 mRNA for Proband, Twin 1 and Twin 2 fibroblasts relative to the two VHL^WT^ fibroblasts (Fig. 2c). These results suggest alternative splicing, specifically, increased exon 2 skipping as an effect of the mutation. At the protein level, pVHL_160_ was the predominant isoform while pVHL_213_ and pVHL_172_ were also detectable in the fibroblasts (Fig. 2d). Consistent with downregulated E1E2E3 transcript, pVHL_213_ was downregulated in mutant fibroblasts compared with WT fibroblasts (Fig. 2d). Contrary to our expectation, pVHL_172_ was also downregulated in mutant fibroblasts (Fig. 2d).

## Discussion and conclusions

Our study shows that the synonymous *VHL* mutation c.414A > G can induce pediatric retinal HGB in the absence of PHEO. In the adult proband, the mutation caused the development of HGB with PHEO. This mutation is a recurrent synonymous mutation in VHL disease. Based on this and two additional recent studies [17, 18], the mutation has been reported in 29 individuals of 8 independent families (**Supplementary Table** **S2**). The overall association is with Type 2A (i.e. diagnosis of PHEO and HGB but rarely RCC). Although this variant was shown to be associated with PHEO [17, 18], the clinical information on its role in HGB was limited. In the clinic, HGB instead of RCC is the major contributor to the unfavorable overall survival of VHL patients [20], highlighting the importance of understanding the etiology of HGB. In this report, the natural history indicates that this variant can be causal for assertive development of HGB in brain and retina needing multiple interventions, strongly recommending that affected asymptomatic patients undergo regular brain, spine and abdominal MRI screenings and ophthalmologic exams.

This is the first report on detecting and validating a synonymous *VHL* mutation using patient-derived fibroblasts. The mutation c.414A > G translates to p.Pro138Pro, yet it is not functionally silent, because the mutation causes aberrant splicing by skipping exon 2. The reduced but not completely abolished pVHL protein in a LOH genetic backdrop may underlie the etiology of VHL disease. Previous studies used lymphoblastoid cell lines established from VHL patients [17, 21], which require transformation of B lymphocytes with Epstein-Barr virus and the immunosuppressant Cyclosporine A [22, 23]. In contrast, the culture of primary fibroblasts is technically straightforward [19] and does not require transformation, a procedure that might complicate the interpretation of the biological functions of tumor suppressor genes to study [24].

Using the fibroblasts derived from three patients and two healthy individuals, we confirmed that the c.414A > G mutation led to *VHL* exon 2 skipping and generated less E1E2E3 but more E1E3, consistent with previous reports [17, 18]. Mechanistically, c.414A > G mutation may dysregulate the exonic splicing enhancer in exon 2 and cause exon 2 skipping [17]. To our surprise, we observed lower protein levels for both pVHL_213_ (encoded by E1E2E3) and pVHL_172_ (encoded by E1E3) in mutant fibroblasts compared with wild type fibroblasts. This result strengthens a similar finding using lymphoblastoid cell lines carrying the c.414A > G mutation [17] and suggest that there may be unidentified mechanisms regulating the translation or protein stability of pVHL_172_ so that this isoform level remains in proportionally lower abundance relative to the combined pVHL_213_ and pVHL_160_ level. It is likely that post-translational mechanisms of negative feedback nature exist to control pVHL_172_ level. Recently pVHL_172_ was identified to possess oncogenic activity when overexpressed in the *VHL*-null RCC cell line 786O [16]. However, since pVHL_172_ level is not increased despite elevated E1E3 transcript level, its contribution to PHEO and HGB is expected to be limited.

The findings from this study and others [17, 18], strongly advocate changing the status of *VHL* c.414A > G variant from “Uncertain significance” to “Pathogenic” for VHL disease in human variant databases (e.g. ClinVar). An unresolved issue is the mechanism how the exon 2 skipping mechanism causes the Type 2A disease phenotype which typically involves missense mutations. Clinically, the PHEO tumors in patients carrying c.414A > G mutation lost the other WT allele [17, 18], demonstrating LOH as described in classic VHL disease [25]. Therefore, it is conceivable that this hypomorphic mutation in the backdrop of LOH creates a residual amount of pVHL activity that makes cells in the adrenal glands and CNS, but not kidney, susceptible to tumorigenesis. Our findings by combing imaging reports and molecular evidence from skin fibroblasts highlight the need to include splicing-altering synonymous mutations into the screening for VHL disease.

## Supplementary information

**Additional file 1 Table S1.** A concise timeline from the proband’s clinical record and natural history of VHL disease. **Table S2.** Summary of clinical cases with synonymous *VHL* mutation c.414A > G, p.Pro138Pro.

## Data Availability

The datasets generated and analysed during the current study are not publicly available because it is possible that individual privacy could be compromised but are available upon reasonable request with fulfillment of Materials Transfer Agreement and in a format compliant with Health Insurance Portability and Accountability Act (HIPAA). To request the datasets, please contact the corresponding authors (K.H. khaldar@nd.edu or X.L. xlu@nd.edu).

## Abbreviations

CNS: Central nervous system
DMEM: Dulbecco’s modified eagle medium
HGB: Hemangioblastoma
HIF: Hypoxia-inducible factor
LOH: Loss of heterozygosity
MRI: Magnetic resonance imaging
PHEO: Pheochromocytoma
RCC: Renal cell carcinoma
RT-PCR: Reverse transcriptase polymerase chain reaction
VHL: Von hippel-lindau
WT: Wild type
