# Supplementary material for: Case report: a synonymous VHL mutation (c.414A > G, p.Pro138Pro) causes pathogenic familial hemangioblastoma through dysregulated splicing
Source: BMC Med Genet. 2020 Feb 27;21:42. doi: 10.1186/s12881-020-0976-7 (PMC7045488; doi:10.1186/s12881-020-0976-7)
Supplement: Supplementary file 1 — Additional file 1 Table S1. A concise timeline from the proband’s clinical record and natural history of VHL disease. Table S2. Summary of clinical cases with synonymous VHL mutation c.414A > G, p.Pro138Pro. [file 12881_2020_976_MOESM1_ESM.pdf]

Supplementary Information

A synonymous *VHL* mutation (c.414A>G, p.Pro138Pro) causes pathogenic familial hemangioblastoma and pheochromocytoma through dysregulated splicing

Fang Liu, Barbara Calhoun, Md. Suhail Alam, Miaomiao Sun, Xuechun Wang, Chao Zhang, Kasturi Haldar, Xin Lu

Table S1. A concise timeline from the proband's clinical record and natural history of VHL disease.

DATE	Test/Surgery	RESULT
**08.22.2003**	VHL Testing	Results + for VHL c.414A>G p. Pro138Pro ; Proband asymptomatic
**10.2.2008**	MRI SPINE<br>MRI BRAIN<br><br><br><br>MRI ABDOMEN/PELVIS	Small enhancing lesions- HGB@ C2, T12, L1 and L2<br>Multiple lesions (9) within posterior fossa- Right Cerebellum: 9mm and 17mm lesions; Left Posterior Cerebellum 4 mm ill-defined enhancement with mild edema and downward displacement of right cerebellar tonsil<br>Left Adrenal Gland mass-consistent with PHEO; mild filling defect right ureter
**10.2008**	SURGERY	Left Adrenalectomy
**01.2009**	SURGERY	Gamma Knife Radiotherapy to cerebellar lesions
**06.2009**	MRI SPINE<br>MRI BRAIN	Stable lesion mid C2 (3.8mm), unchanged,L1,L2<br>After gamma knife the two larger right cerebellar lesions(from 10/2008) now one large lesion 22x25x28; increased vasogenic edema and increased mass effect on inferior 4th ventricle; development of hydrocephalus
**10.2009**	MRI Abdomen/Pelvis	S/P left adrenalectomy-no evidence of recurrent tumor
**11.2009**	SURGERY	Craniotomy Posterior Fossa; Resection of large HGB
**12.2009**	MRI BRAIN	Slight increase in size of previously treated right cerebellar lesion; physicians to monitor Postop MRI area of craniotomy stable with complete resection of tumor and resolution of edema and hydrocephalus;
**2010**	MRI SPINE<br><br>MRI BRAIN<br>MRI ABDOMEN/PELVIS	Stable, leptomeningeal enhancements at C2 and L1, L2 unchanged; mild to moderate degenerative changes L5 to S1 more prominent than prior MRI<br>Cranial lesions stable<br>S/P adrenalectomy; Hyper intensity noted at lower pole of right kidney-too small to characterize
**2011**	MRI SPINE<br>MRI BRAIN<br>MRI ABDOMEN/PELVIS	No changes in MRI of Spine, Brain or Abdomen/Pelvis from prior MRI<br>Nodules in cerebellar hemisphere unchanged; no new nodules
**2012**	MRI SPINE<br>MRI BRAIN<br>MRI ABDOMEN/PELVIS	Slightly thicker plaque are dorsal cervical spine at C2 (5mm,prior study measured 3mm)<br>Right medial cerebellar lesion associated with mild edema but no mass-effect; no other changes<br>Hyper intense focus at the lower pole of right kidney unchanged
**2013**	MRI SPINE, BRAIN, ABDOMEN/PELVIS	No new MRI findings from 2012; no changes
**2014**	MRI SPINE<br>MRI BRAIN<br>MRI ABDOMEN/PELVIS<br>XRAY, MRI KNEE	Dorsal C2 enhancement 4mm unchanged;<br>Stable; no change from 2013<br>Stable T2 hyper intensity lateral aspect of lower right kidney<br>Knee pain; small patellar spur noted otherwise normal(X-ray)<br>MRI revealed moderate medial joint arthrosis; Patello-femoral chondromalacia
**2015**	MRI SPINE	Unchanged cervical spine lesions; mild degenerative changes cervical spine; small foci of leptomeningeal enhancement dorsal cord of C2, the conus of L1,L2 and several cauda equine nerve roots

**2016**	MRI SPINE,BRAIN, ABDOMEN/PELVIS	NO change from prior year
**2017**	Xray Knees	Patellofemoral joint degenerative changes
**2018**	MRI SPINE<br><br><br>MRI BRAIN<br>MRI ABDOMEN/PELVIS	Small focus of leptomeningeal enhancement along dorsal cord of C2 unchanged; multiple small foci of leptomeningeal enhancement along the conus and cauda equine nerve roots unchanged<br><br>6 sub centimeter lesions unchanged in posterior fossa; no evidence of disease progression<br>Unchanged kidney hypertense focus right lower

Table S2. Summary of clinical cases with synonymous *VHL* mutation c.414A>G, p.Pro138Pro.

**Family ID**	**Lenglet, 2018 Family 11**	**Lenglet, 2018 Family 12**	**Flores, 2019 Family 1**	**Flores, 2019 Family 2**	**Flores, 2019 Family 3**	**Flores, 2019 Family 4**	**Flores, 2019 Family 5**	**Present study, Family 1**
Number of affected individuals in family	3	4	7	5	3	3	1	3
Age at onset of proband (years)	na	na	32	53	31	20	47	47
Age range of diagnosis of affected individuals (years)	na	na	12-73	29-64	31-50	20-27	47	6-47
Gender of affected individuals (F:M)	1:2	3:1	2:5	1:4	0:3	0:3	1:0	2:1
VHL Related phenotype(#of affected individuals)	PHEO 3 HGB 2 RCC 1	PHEO 4	PHEO 7 HGB 1/7 Renal Cyst 1/7	PHEO 5 PGL 1/5 HGB 1/5	PHEO 3	PHEO 3 HGB 1/3	PHEO + PGL (1)	HGB 3/3 PHEO 1/3
Initial Disease Manifestation	?	PHEO	PHEO	PHEO	PHEO	PHEO	PHEO	HGB PHEO
# of individuals with multiple PHEO (% affected in family)	?		0 0%	3 (60%)	0%	2 (67%)	1(100%)	0 (0%)
Malignancy	Yes renal	na	no	Yes-lung mets	no	no	no	no
Other VHL Manifestations	paragan glioma	no	Spinal HGB	Spinal HGB	no	Spinal HGB	no	Spinal HGB Retinal HGB
VHL disease subtype			2A	2A	2C	2A	2C	2A
Other manifestations (# of individuals)		no	Thyroid Nodule-1 Bladder Carcinoma-1	no	no	Parathyroid nodule (1) Esophageal carcinoma (1)	no	no
Followup time	na	na	30 years	18 years	10 mos	25 years	20 years	10 years
